# Supplementary material for: Characterizing the Anoxic Phenotype of Pseudomonas putida Using a Bioelectrochemical System
Source: Methods Protoc. 2019 Mar 30;2(2):26. doi: 10.3390/mps2020026 (PMC6632142; doi:10.3390/mps2020026)
Supplement: Supplementary file 1 [file mps-02-00026-s001.pdf]

Supplementary material for Characterizing the anoxic phenotype of *Pseudomonas putida* using a bioelectrochemical system

Bin Lai [1,2], Anh Vu Nguyen [1] and Jens O Krömer [1,2,*]

1 Systems Biotechnology group, Department of Solar Materials, Helmholtz Centre for Environmental Research – UFZ, 04318 Leipzig, Germany
2 Center for Microbial electrochemical Systems (CEMES), Advanced Water Management Center, the University of Queensland, Brisbane QLD 4072, Australia
* Correspondence: jens.kroemer@ufz.de; Tel.: +49-341-235-4688

Figure S1	Cyclic voltammetry characterization of carbon cloths with different pre-treatment methods.
Figure S2	Growth property of *Pseudomonas putida* F1 in shaking flasks with different gas-permeable stoppers in minimum mineral medium.

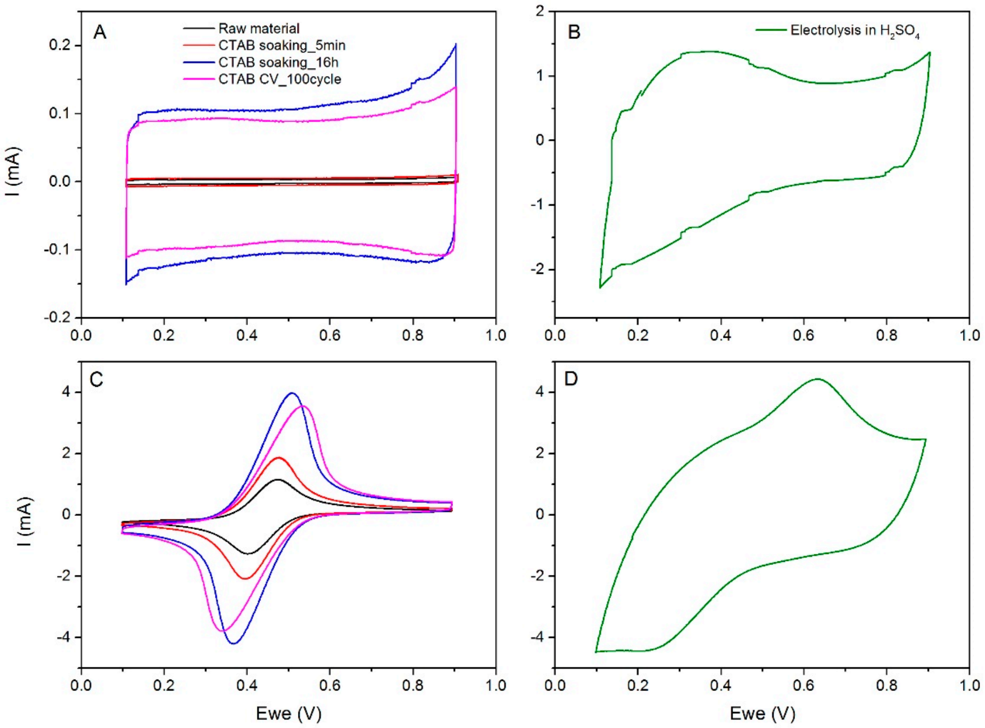

Figure 1. Cyclic voltammetry characterization of carbon cloths with different pre-treatment methods. A and B present the test conditions of 100mM KCl and 50 mV/s scanning rate, while C and D give the results under the conditions of 1mM ferricyanide, 100 mM KCl and 50 mV/s scanning rate. Working electrode potentials (E_{we}) are reported versus SHE.

The raw carbon cloth was ordered from Fuel Cell Store (1071 HCB), and has a hydrophobic surface property. Two pre-treatment methods were tested to improve its hydrophilicity: Cetrimonium bromide (CTAB) based method [1] and electrolysis in acids solution [2]. The original protocols from the literatures were tested, presented in the figure as *CTAB soaking_5min* and *Electrolysis in* H_2SO_4, as well as two more modified CTAB methods, termed *CTAB soaking_16h* and *CTAB CV_100cycle* where the former one was soaking the carbon cloth in 2 mM CTAB solution for overnight (~16 h) and the latter was anodic polymerization of CTAB on the carbon cloth using cyclic voltammetry under the following conditions: potential range of [−0.6, 1.2] V vs SHE, scanning rate of 50 mV/s, 2 mM CTAB solution in 100 mM KCl solution.

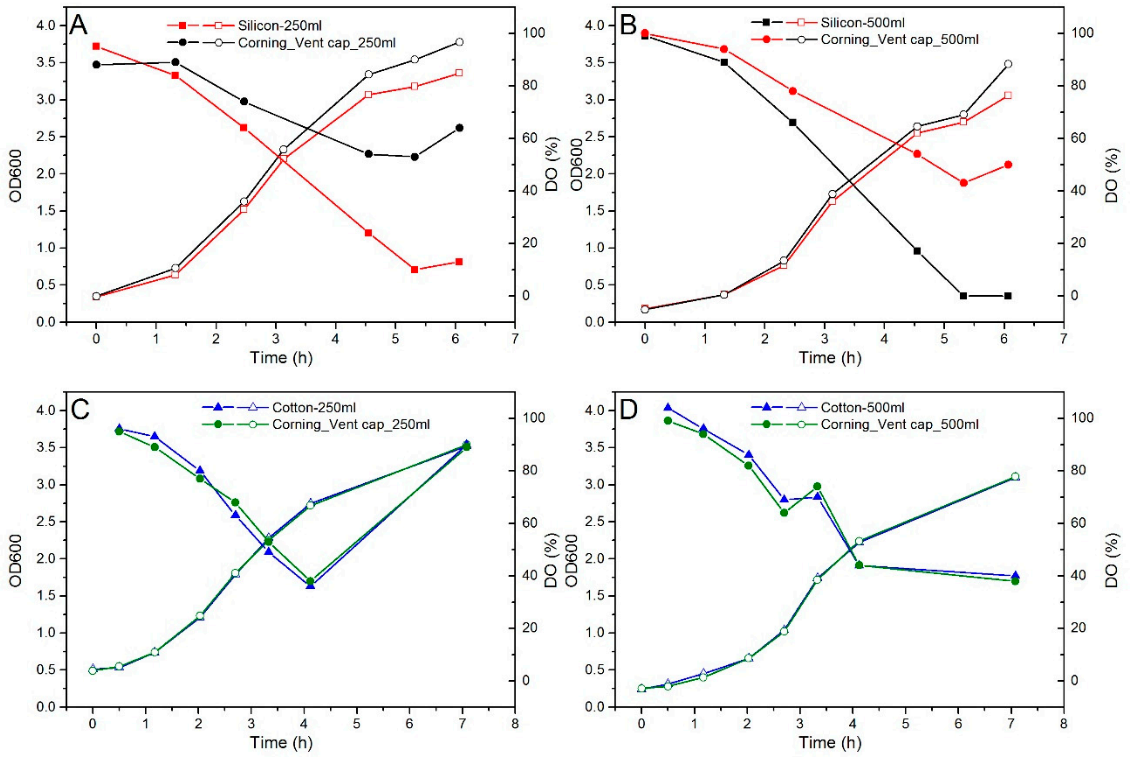

Figure 2. Growth property of *Pseudomonas putida* F1 in shaking flasks with different gas-permeable stoppers in minimum mineral medium.

The shaking flasks used for all conditions were the Corning® disposable Erlenmeyer flasks (#431407 for 250 ml size and #431401 for 500 ml size). The original membrane vent caps (labelled as *Corning-Vent cap_* in the figure) were tested as a standardized control. The cotton stopper (labelled as *Cotton-* in the figure) was made locally by wrapping medical cotton balls with gas permeable gauze, and the silicone foam stoppers with sand core filter (labelled as *Silicon-* in the figure) were ordered from China (Nantong, JiangSu, China). Two sizes of flasks were tested, with maximum volume of 250 ml and 500 ml respectively, as labelled in the figure. And 10% of the maximum volume medium were filled in each flask to maximize the gas transfer from gas phase to liquid phase inside the flasks. The test conditions were: wild type *Pseudomonas putida* F1, DM9 medium with 5 g/L glucose as sole carbon source, 30 °C and 200rpm in shaking incubator (Infors HT, Switzerland). The dissolved oxygen (DO) was measured using a two-point calibrated optical oxygen sensor (OXY-4 mini, Presens, Germany) with sensor spots (SP-PSt3, Presens, Germany) attached to the inner surface of Erlenmeyer flasks.

Reference

1. Guo, K.; Soeriyadi, A.H.; Patil, S.A.; Prévoteau, A.; Freguia, S.; Gooding, J.J.; and Rabaey, K. Surfactant treatment of carbon felt enhances anodic microbial electrocatalysis in bioelectrochemical systems. *Electrochem. Commun.* **2014**, *39*, 1–4.
2. Tang, X.; Guo, K.; Li, H.; Du, Z.; Tian, J. Electrochemical treatment of graphite to enhance electron transfer from bacteria to electrodes. *Bioresour. Technol.* **2011**, *102*, 3558–3560.
